# 

**Published:** 1898-08-13

**Authors:** 


**Notices of Births, Deaths, and Marriages** are inserted in this column at a charge of 2s. 6d. for an announcement not exceeding four lines.

### NOTICE TO CORRESPONDENTS.

**All MS., Letters, Books for Review, and other matters intended for the Editor** should be addressed The Editor, "The Hospital," The Hospital Building, 28 & 29, Southampton Street, Strand, W.C.

**The Editor cannot undertake to return rejected MS., even when accompanied by stamped directed envelope.**

**Notice to Advertisers and Subscribers.**

**All Advertisements,** Orders for Copies of The Hospital, and Business Communications should be addressed to **THE MANAGER, (Not to The Editor),** The Hospital, The Hospital Building, 28 & 29, Southampton Street, Strand, London, W.C.

**The Cost of Subscription,** post free, is as follows:—Per week, 2½d. per quarter, 2s. 8d.; per half-year, 5s. 3d.; per annum, 10s. 6d. Foreign:—Per annum, 15s. 2d.; payable, in all cases, in advance.

**NOTICE.—Vol. XXIII. of "The Hospital" (October, 1897, to March, 1898), in handsome cloth binding, is now on sale at the Office of this Journal, price 7s. 6d. Cases for binding the Numbers contained in this Volume, 1s. 6d. Index to Vol. XXIII., 2½d., post free.**

**The Monthly Part for August is now ready. Price 6d., by post 8d.**

### BOOKS RECEIVED.

H. K. Lewis.

"The Extra Pharmacopœia," by William Martindale, F.L.S., F.C.S.; with Medical References and a Therapeutic Index, by W. Wynn Westcott, M.B.Lond.

The Central Publishing Co.

"The Dictionary of Diseases."

The Scientific Press.

"The Care of Consumptives." By W. H. Daw, L.R.C.P.

Digby, Long, and Co.

"Memoirs of a Young Surgeon." By Frederick Ashurst, M.B.

Chas. J. Thynne.

"Short Sermons for Sick Rooms." By the late Josiah Bateman, M.A.

Swan Sonnenschein and Co.

"Natural Hygiene." By H. Lahmann, M.D.

F. A. Davis Co.

"Conservative Gynæcology and Electro-Therapeutics." By Y. Betton Massey, M.D.

### Scraps and Gleanings.

The Metropolitan Radical Federation have condemned street collections, stating that, in their opinion, they are a public nuisance.

The total number of patients at the Birmingham Children's Hospital during the half-year ending June 30th was 6,931, against 6,?74 during the first half of 1897.

The Newcastle Hospital Sunday Collection for 1897 amounted to £4,584 (or, deducting expenses, to £4,2 3), a falling off of £155 as compared with last year.

The Ladies' Committee of the Hastings and St. Leonards Hospital have decided to make a further house-to-house collection on behalf of the hospital this year.

The Haywood Hospital at Burslem has received a donation of £300 from Miss S. B. Maddock (sister to the chairman) in memory of her late brother, Mr. Thomas Maddock.

As showing the demand for admittance to the Royal Mineral Water Hospital at Bath, the report mentions that there were on May 1st last 145 persons waiting for vacancies.

We regret to note that the receipts from annual subscribers at the Hereford General Infirmary are not increasing in a fair proportion to the increase in the patients and the expenditure.

The ordinary revenue of the Northern Infirmary at Inverness for 1897 amounted to £3,799, or £286 more than 1896. The ordinary expenditure amounted to £3,955, or an increase of £98.

The total cost of building and furnishing the new wards at the Warneford Hospital, Leamington, will be not less than £10,000, of which the building of the new wing will amount to about £6 760.

At the house-to house collection made by the Dover Amalgamated Friendly Society on behalf of the Dover Hospital the sum of £74 odd was collected, in addition to £40 odd collected in the streets.

The Ropner Convalescent Home at Middleton-St.-George, which was recently opened by the Lord Bishop of Durham, is the gift of Colonel and Mrs. Ropner, in memory of the Queen's Diamond Jubilee.

The special general meeting of the trustees to discuss the question of rebuilding the Manchester Royal Infirmary has been postponed until the autumn in order that a fully representative meeting shall be secured.

Plans for the new accident wards at the Devonshire Hospital, Buxton, have been prepared. It is proposed to provide two additional wards, one for three beds and one for five. The cost will be from £1,200 to £1,400.

Mr. H. Studt, who has for many years past given a benefit in aid of the funds of the Swansea Hospital, has been doing the same in aid of the Leominster Cottage Hospital, which it is hoped will soon become *un fait accompli*.

The Royal Victoria Hospital, Belfast, is now on the way to completion, and the committee express themselves as very satisfied with the way the subscriptions have come in. They have now in the bank a sum of £64,000 to the credit of the fund.

One hundred and forty-nine more in-patients were treated at the Devonshire Hospital, Buxton, during 1897-8 than in the previous year, the numbers being 2,919 and 2,770 respectively. Two hundred and forty-four out-patients were admitted during the twelve months.

The Jubilee collection at Oldham in aid of the infirmary has reached the gratifying total of £7,972, which, less the expenses, leaves a net balance of about £7,830. At the same time, much additional accommodation is required both for general cases and for the special departments.

Londoners are apt to forget the enormous amount of work done by their hospitals, and need to be continually reminded of this fact, which is to some extent brought out by figures. During 1896 the number of patients treated at hospitals reached the enormous total of nearly two millions.

An institution, by name "The Home for Worn-out, Aged, and Destitute Women," is missing. A testator has bequeathed the sum of £ [illegible]00 to an institution of this name. Inquiries have elicited the fact that many years ago a charity under this title was in existence, but all trace of it has now disappeared.

The income at the Northampton General Infirmary in 1896-7 was £7,768, and the expenditure £8,621; in 1895-6 it was £8,044, and the expenditure £8,621. It is estimated that there will be a considerable loss of subscriptions during the current year, owing to the establishment of the Kettering Hospital.

The Eye Infirmary at Newcastle, which is the oldest ophthalmic institution in the Northern counties, has been appealing for an increase in income to the extent of £300 a year. The ordinary receipts for 1897 were £527, and the expenditure £799, leaving a debit balance at December 31st, 1897, of £565, to partly meet which £500 was taken from invested capital.

The Abergavenny Workmen's Saturday Hospital Fund have decided to present the local cottage hospital with £25. Last year they gave £30, and the reason for the decrease in the grant this year is that the majority of the committee wish to keep some funds in hand in view of the possibility of the fund being appealed to on behalf of the united dispensary and hospital which it is hoped shortly to establish.

The Bournemouth Town Council have had brought before their notice the question of the abolition of the fees at the Sanitary Hospital The opposers of this change argue that such abolition would be illegal, and quote a certain section of the Public Health Act in support of this contention. The public auditor of Bournemouth has to answer for this discovery, so he is evidently trying to justify his appointment, seeing that under the Municipal Corporations Act a public auditor is not required. Bournemouth, however, has thought differently, and it appears likely that they will continue to hold a difference of opinion with regard also to the question under consideration, viz , the abolition or not of the fees at the Sanitary Hospital.

### The Student of Medicine.

**APPOINTMENTS.**

Dr. F. H. H. Elvins, appointed Resident Surgeon at the Ararat Hospital, Victoria. H. B. Gardner, M.R.C S.Eng., L.R.C.P.Lond., appointed Anæsthetist to Charing Cross Hospital. C. G. Gooding, B.A., M.B., B.C.Cantab., appointed Honorary Surgeon to the Royal Kent Dispensary. C. J. Heath, F.R.C.S.Eng., appointed Surgeon to the Throat Hospital, Golden Square, W. P. T. Herring, M.B., C.M., appointed House Surgeon to the Edinburgh Royal Maternity and Simpson Memorial Hospital. E. L. Hickey, M.D.Dub., M.R.C.S.Eng , appointed Government Medical Officer and Vaccinator at Nyngau, New South Wales. E. B. Hicks, L.R.C.P.Lond., M.R.C.S.Eng., appointed Medical Officer for the Easingwold District and the Workhouse of the Easingwold Union. C. E. Hollings, L.R.C.P., L.R.C.S.Edin., appointed Medical Officer for the Kilham District of the Driffield Union. A. B. How, B.A.Oxon, M.R.C.S., L.R.C.P.Lond , appointed Medical Officer for the Claygate District of the Kingston Union. J. W. Hunt, M.D., appointed Medical Officer for the Seventh District of the Hackney Union. F. Hunton, M.D.Durh., M.B., B.S., appointed Medical Officer of Health to the Sedgefield Rural District Council. J. W. Lloyd, M.R.C.S., L.R.C.P.Lond., L.D.S.Edin., appointed Honorary Surgeon to the Liverpool Dental Hospital. A. N. McArthur, M.R.C.S.Eng., appointed House Surgeon to the General Hospital, Launceston, Tasmania. G. E. Miles, L.R.C.P.Lond., appointed Medical Superintendent at the Hospital for Insane, Rydalmere, New South Wales. C. R. Nicholson, L.R.C.P., M.R.C.S., appointed Senior House Surgeon to the Hastings, St. Leonard's, and East Sussex Hospital. T. J. O'Mera, B.A., M.B., appointed Medical Officer for the Dispensary District of Tullagh in the Skibbereen Union. S. J. Richards, M.B., appointed Medical Officer at Mount Morgan, Queensland. J. A. Ross, M.B., C.M.Aberd., appointed Medical Officer to the Penistone Union Workhouse. A. Routh, M.D.Lond., appointed Obstetric Physician to Charing Cross Hospital. J. C. Sibley, M.D New York, appointed Medical Officer of the Goodooga Hospital, New South Wales. G. F. B. Simpson, M.B., C.M., appointed House Surgeon to the Edinburgh Royal Maternity and Simpson Memorial Hospital. C. H. Stewart, M.B., D.Sc., F.R.S.E., appointed Professor of Public Health and Sanitary Science at Edinburgh University. W. E. Smith, M.B., C.M.Edin., appointed Junior House Surgeon to the Royal London Ophthalmic Hospital, Moorfields, E.C. J. O. Symes, M.D.Lond , appointed Registrar to the Royal Hospital for Sick Children and Women, Bristol. St. Clare Thomson, M.D., M.R.C.P.Lond., F.R.C.S.Eng., appointed Physician to the Throat Hospital, Golden Square. L. T. Thorne, M.D., appointed Medical Examiner to the Technical Educational Board of the London County Council. M. Thorne, L.S.A.Lond., M.D.Brux , appointed Clinical Assistant to the Royal Free Hospital. C. M. Vernon, appointed Medical Officer of Health for Ashford Urban District. Dr. W. J. Vincent, appointed Second Assistant Medical Officer to the Wadsley Asylum. A. Whitfield, M.D.Lond., M.R.C.P., appointed Physician to the Skin Department, Great Northern Central Hospital. L. A. Williams, M.B., M.S , appointed Visiting Surgeon to the Chester General Infirmary. W. C. Williamson, M.D., appointed Medical Superintendent to the Hospital for Insane, Gladesville, New South Wales. Dr. T. Wilson, appointed Medical Officer to the Parish of Birmingham Infirmary. H. W. A. Sandell, L.R.C.P. Edin., M.R.C.S.Eng., appointed Medical Officer of Health to the Linslade Urban District Council W. T. H. Spicer, M.A., M.B.Camb., F.R.C.S.Eng., Ophthalmic Surgeon to the Metropolitan Hospital and to the Victoria Hospital for Children, appointed Assistant Surgeon to the Royal London Ophthalmic Hospital, Moorfields, E.C. C. Stain, M.B., C.M.Edin., appointed Medical Officer for the Audlem District of the Nantwich Union. F. G. Stewart, M.R.C.S Eng., L.S.A., appointed Medical Officer of Health to the Wadebridge Urban District Council. E. F. Syrett, M.D Durh , M.R.C.S.Eng., appointed Medical Officer and Public Vaccinator for the Eighth District of the Lexden and Winstree Guardians. S. G. Toller, M.D., M.R.C.P.Lond., M.R.C.S.Eng., appointed Professor of Clinical Medicine at the School, and Physician to the Kasr-el-Aini Hospital. O. B. Trumper, M.B., Ch.B., appointed Medical Officer for the Market Rasen First District and the Tealby District of the Caistor Union.

28 & 29, SOUTHAMPTON STREET, STRAND, LONDON, W.C.

